# Pathways for accelerated bacterial spore killing with ohmic heating

**DOI:** 10.1038/s41538-025-00537-1

**Published:** 2025-08-07

**Authors:** Shyam K. Singh, Chaminda P. Samaranayake, George Korza, Mohamed M. Ali, Peter Setlow, Sudhir Sastry

**Affiliations:** 1 The Ohio State University, Columbus, OH USA; 2 University of California, Davis, CA USA; 3 UCONN Health, Farmington, CT USA

**Keywords:** Chemical engineering, Bacteria, Reaction kinetics and dynamics, Biological physics

## Abstract

The mechanism by which ohmic heating (OH) accelerates bacterial spore killing compared to conventional heating (CH) is unclear. This study used genetically modified *Bacillus subtilis* spores to investigate OH’s impact on specific components. Flow cytometry assessed membrane integrity, and molecular dynamics (MD) simulations examined the DNA-SASP complex under an electric field. Among the inner membrane (IM) proteins (YetF, YdfS, and YkjA) tested for its resistance against OH and CH, YeTF was found to be the most significant contributor to spore resistance for both treatments. SASP, SpoVA proteins, and Ca-DPA interacted with the field, showing specific effects at certain temperature and field intensity combinations. Flow cytometry showed spore staining with propidium iodide (PI), which increased with higher field intensities, indicating significant IM damage. MD simulations showed that the electric field caused the SASP-DNA complex to dissociate, with greater separation at higher field intensities. Thus, OH accelerates spore killing by affecting key IM and core molecules.

## Introduction

Food is essential to sustain life on earth, but consuming contaminated food can cause severe illness and, in some instances, may result in death. Despite advances in food safety assurance, according to a report by FAO^1^, every year, 1 in 10 people get sick, and 420,000 die annually from consuming contaminated food, with an economic impact estimated at $110 billion annually. Bacteria contribute to almost 38% of these diseases in the USA^2^. We focus herein on bacterial spores, some of the most resistant life forms on earth. Spores formed by *Bacillus* and *Clostridium* species are extremely resistant to various treatments, including dry and wet heat, gamma and UV radiation, desiccation, and oxidizing agents^3,4^, and are of concern due to pathogenicity^5^ and as agents of food spoilage (*Bacillus coagulans*, *Clostridium butyricum*, *Clostridium sporogenes* etc.).

The food industry currently inactivates sporeforming organisms (particularly *Clostridium botulinum*) using intense heat, which can cause the food to lose some of its essential nutrients and color^6^. None of the purely nonthermal methods investigated (high pressure, pulsed electric fields) have been successful against bacterial spores. In this context, ohmic, or Joule-effect heating has been exceptionally successful, not only due to its capability for uniform internal heat generation in food materials, but also in accelerating inactivation of sporeformers when maintaining temperature histories identical to conventional heating (CH)^7–11^. However, the reasons for accelerated inactivation are unclear and need further elucidation.

Spores derive much of their resistance from their unique structure, which includes low core water content, reduced inner membrane (IM) permeability, and the saturation of spore DNA with α/β-type small acid-soluble proteins (SASP), among other factors^3^. Given that spores contain minerals and polar molecules, we hypothesize that these molecules respond to an externally applied electric field, potentially explaining the accelerated killing^12,13^. To determine whether ohmic heating (OH) targets specific spore components, we used several mutants of *Bacillus subtilis* spores and compared their inactivation kinetics with wild-type spores, to identify if a component is targeted by the applied field. Additionally, we performed flow cytometry with fluorescent dyes to study the integrity of the spore’s IM. To further understand the interaction of the electric field with key spore components at the molecular level, we conducted molecular dynamics simulations. Together, these analyses will help us identify the most likely pathways through which OH accelerates the killing of spores.

## Results

### Effects of mutant spores

**Fig. 1:**
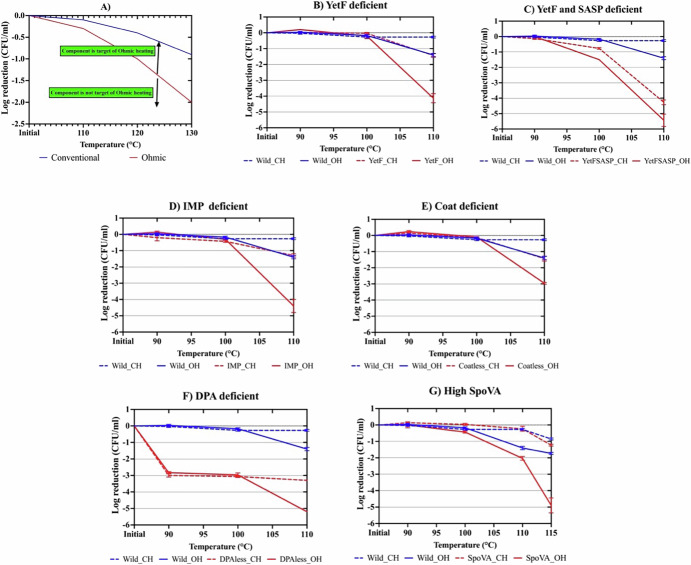
Inactivation of spores of *B. subtilis* lacking essential components under OH and CH conditions. **A** Rationale for using different mutant spores and log reduction in populations of spores of *B. subtilis* **B** YetF-deficient (PS4550), **C** YetF and SASP deficient (PS4551), and **D** YetF, YdfS, and YkjA (IMP) deficient (PS4531), **E** coat-deficient (PS4150), **F** DPA-deficient (PS3406), and **G** spores containing high levels of the SpoVA operon, all after OH treatment at different temperatures (90, 100, and 110 °C), or in CH treatment by following the heating rate with OH 30 V/cm. OH- Ohmic heating, CH- Conventional heating.

We used various *B. subtilis* mutants, each missing a specific structural component, to understand why ohmic treatment inactivates spores more effectively and quickly. By comparing OH and CH inactivation profiles of wild-type and mutant spores, we identified OH-specific targets. If a component’s removal reduces the OH-CH killing difference, it suggests that component is susceptible to OH, as its absence eliminates the electrical effects seen in wild-type spores (Fig. 1A). We investigated a mutant lacking the IM YetF protein, which increases spore IM rigidity. At 90 and 100 °C (non-lethal for *B. subtilis*), no significant difference (*P* > 0.05) was observed between OH and CH treatments for both wild-type and YetF-deficient mutant PS4550. However, at 110 °C, OH resulted in significantly higher (*P* < 0.05) spore inactivation than CH, with inactivation (logCFU/ml) over triple for both wild-type (0.30 ± 0.08 with CH vs. 1.40 ± 0.08 with OH) and YetF-deficient spores (1.00 ± 0.07 with CH vs. 4.50 ± 0.28 with OH) (Fig. 1B). Similar results were seen with spores lacking 3 out of 5 YetF homologs, including YetF itself and its less abundant homologs, YdfS and YkjA, strain PS4531, indicating that YdfS and YkjA proteins are not major contributors to spores’ resistance to CH or OH (Fig. 1D).

Our examination of spores lacking both YetF and SASP revealed intriguing findings. Previously, we identified SASP as an OH target, shown by the convergence of OH and CH curves for the PS578 mutant lacking SASP^11^. In this study (Fig. 1C), the absence of both SASP and YetF (PS4551) reduced the difference in inactivation between OH and CH from 366% (*P* < 0.05) for the wild type to 25% (*P* > 0.05). Additionally, spores lacking both SASP and YetF showed reduced resistance to OH and CH at 100 °C (*P* < 0.05), unlike those lacking only SASP^11^. The absence of YetF increases IM permeability, allowing easier electric passage during OH, causing more core damage. Across all strains, OH effects were more pronounced at the highest temperature, highlighting a synergistic relationship between temperature and electricity in spore killing^10,11,14,15^.

We tested mutants lacking coat (PS4150) and DPA (PS3406), and spores with higher SpoVA levels (PS3411). Spores without the coat showed a similar inactivation trend to wild-type spores, with only a slight decrease in resistance for CH (*P* > 0.05) and a significant decrease in resistance to OH (*P* < 0.05), but the change in resistance was not as big as PS4551 and PS4550. However, while the coat’s absence slightly reduced resistance to OH and CH, the difference in killing between OH and CH persisted (*P* < 0.05), indicating the coat does not interact with electricity. Spores lacking DPA, crucial for reducing core water content and spore resistance^4,16^, showed significantly higher inactivation (*P* < 0.05) with both OH and CH, even at 90 °C (Fig. 1E). This suggests DPA is essential for wet heat resistance even with OH. The similar killing profiles for OH and CH at 90 and 100 °C imply electricity interacts with DPA. At 110 °C, OH and CH curves diverged, with OH causing higher killing (*P* < 0.05), consistent with other studies showing additional mechanisms under most lethal conditions (either high applied field strengths (FS) or the highest temperature). Finally, we examined mutant spores with an ~fivefold stronger promoter of the *spoVA* operon^16^ that encodes proteins crucial for CaDPA entry during spore formation and, most importantly, for its release during spore germination. Spores of this strain have ~ fourfold elevated levels of SpoVA proteins (↑SpoVA) compared to that in wild type spores. Notably, recent work has found that the ↑SpoVA mutation causes spontaneous germination of spores with a much more fluid IM than normal^17^. Thus, we investigated if ohmic treatment could open the ↑SpoVA strains’ CaDPA channels to release DPA, thus increasing core water content and reducing spore resistance. No OH effect on the strain with ↑SpoVA proteins was seen until 110 °C, but at 115 °C, OH significantly increased these spores’ killing over that by CH), presumably by causing rapid CaDPA release, unlike CH (*P* > 0.05). This supports our hypothesis that some OH effects are evident only under specific conditions (Fig. 1G). Note that 115 °C was used only for this strain because this study continued from a previous study which used this temperature^11^. As we progressed, we found this temperature too lethal for some strains, so we changed to 90, 100, and 110 °C for the other mutants.

### Flow cytometry analysis

**Fig. 2:**
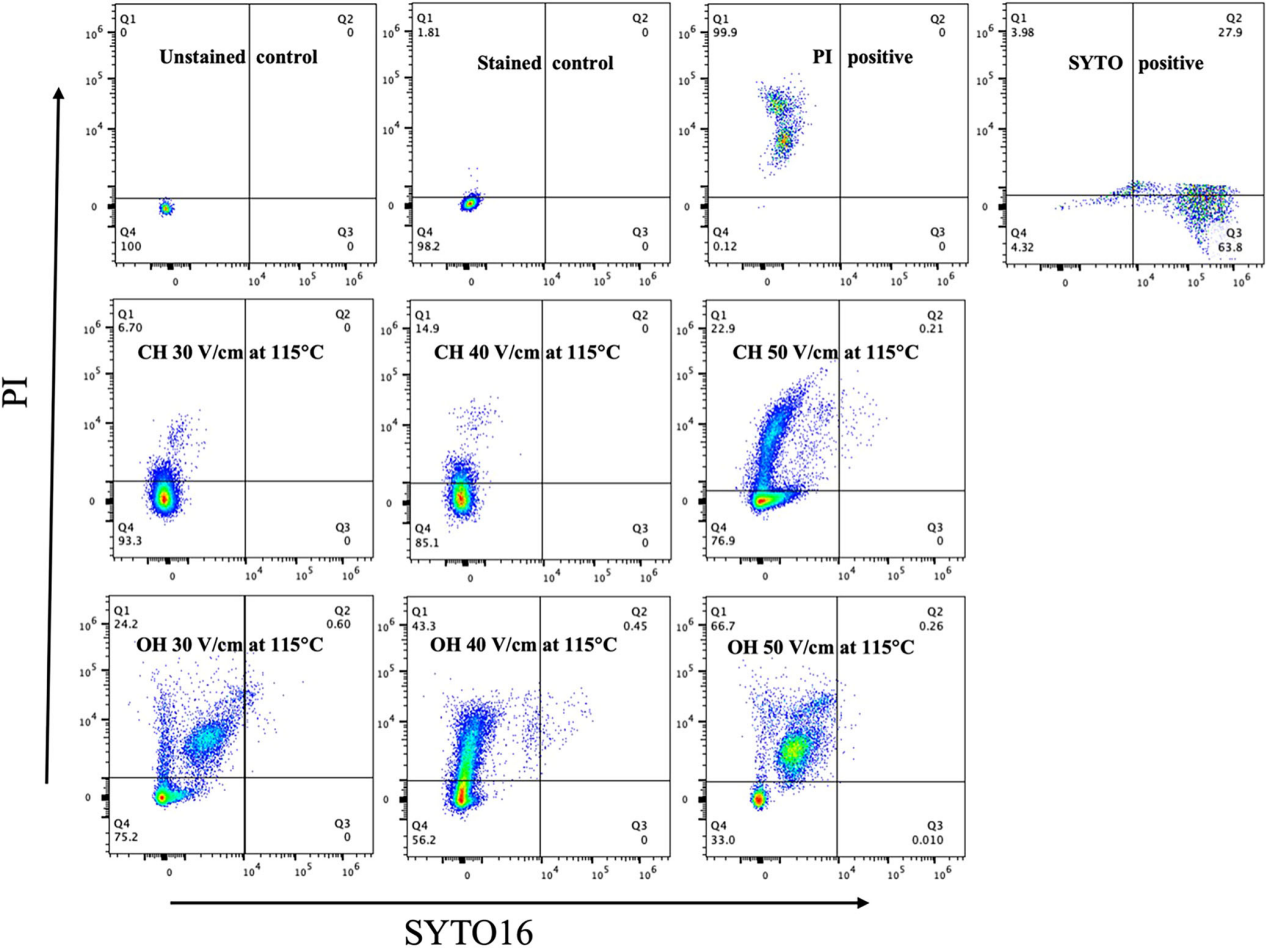
Flow cytometry plots for *B. subtilis* wild-type spores treated with OH and CH at 115 °C under various applied electric field intensities (30, 40, and 50 V/cm). The first line displays the fluorescence signals from the control samples, the second line represents samples treated with CH), and the third line depicts samples treated with OH. The numbers below each quadrant indicate the percentage of spore population in that specific quadrant. Quadrant 1 (Q1) indicates spores stained with PI, Q4 represents the population not stained by either PI or SYTO16, Q3 signifies spores stained by SYTO16, and Q2 represents a population with unknown characteristics.

We used nucleic acid fluorescent dyes propidium iodide (PI) and SYTO 16 to stain bacterial spore DNA. PI can only enter spores if the membrane is compromised or if treatment alters the IM structure, allowing access to the core. Neither dye stains dormant spores, but their staining helps understand IM reactions to treatment. Four controls were used: untreated and unstained (background and sample homogeneity control), untreated and stained (autofluorescence check), PI-positive (spores autoclaved for 60 min), and SYTO-positive (spores germinated with L-alanine following a heat shock) (Fig. 2). The lack of SYTO 16 staining in all but one Q3 panel in Fig. 2 indicates that almost all of these spores have not germinated, as there is no SYTO 16 staining in dormant spores, since saturation of spores’ DNA by SASP blocks SYTO 16’s access to the DNA^18^. Thus, DNAs in spores of all other panels (except the upper right one) are still saturated by SASP and thus have not germinated. However, in the upper right panel in Fig. 2, spores are germinated, SASP are degraded, SYTO 16 binds to spore DNA and gives the fluorescence seen in Q3 in this panel, while spores are not germinated by the treatments in any of the other nine panels of Fig. 2.

The percentage of spores stained with PI increased with higher heating rate associated with high FS for both OH and CH, correlating with inactivation data (greater heating rate led to higher inactivation). OH significantly (*P* < 0.05) stained more spores under specific conditions compared to CH. For instance, CH stained 6.70%, 14.9%, and 22.9% of spores with PI at 30, 40, and 50 V/cm, respectively, with corresponding inactivation of 0.9 ± 0.06, 1.2 ± 0.10, and 2.2 ± 0.06 logCFU/ml. In contrast, OH stained 24.2%, 43.3%, and 66.7% of spores at the same voltages, with inactivation of 1.7 ± 0.04, 3.1 ± 0.14, and 4.7 ± 0.28 logCFU/ml, respectively. These results align with findings from genetically modified spores, suggesting OH increases IM permeability.

### Electric field effects on the DNA protection by SASP

**Fig. 3:**
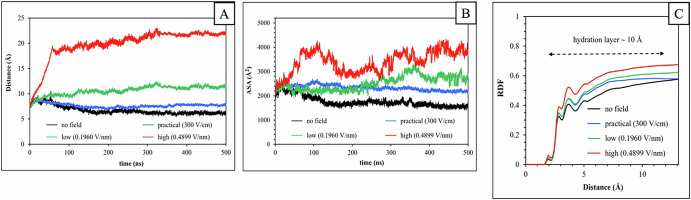
Structural integrity of the DNA-SASP complex in the absence and presence of different electric fields. **A** Center-of-mass distance between the SASP1-2 dimer and DNA. **B** Accessible surface area of the DNA structure and **C** Radial distribution function (RDF) of the water oxygen atom around the DNA in the DNA-SASP complex calculated in the absence and presence of different electric fields. The hydration layer thickness is marked considering approximately 3–5 hydration shells from the DNA’s surface^21^.

SASP binding is crucial for protecting spore DNA from damage, forming a barrier against cleavage by various agents^19,20^. SASP protomers bind tightly around DNA in dimerized units, optimizing their arrangement and interaction with the DNA minor groove^19^. The center-of-mass distance between SASP1-2 dimer and DNA was measured to assess their separation under electric fields (Fig. 3A). Increasing FS lead to greater SASP1-2 dimer-DNA separation, diminishing DNA protection. Profiles comparing no field to practical electric field (300 V/cm) show prolonged field exposure causing significant intermolecular separation, consistent with SASP structural distortions (Supplementary Fig. 1) affecting DNA binding. This separation is driven by the electrophoretic force ( = q × E)^21^, where positively charged SASP ( + 1.6 × 10⁻¹⁹ C) and negatively charged DNA (−3.2 × 10^−^¹⁸ C) move oppositely in the field. Higher field (E) intensifies this force, increasing separations as observed in Fig. 3A. Capillary electrophoresis studies corroborate these findings, demonstrating how electric fields below 1 kV/cm can disrupt DNA-protein complexes^22^. To assess SASP protomer separation, we measured the accessible surface area (ASA) of the DNA structure under electric fields. Normally, 35–40% of the protein and DNA surfaces are buried in the DNA-SASP complex^19^. As protomers separate, this buried surface area decreases, increasing the ASA of the binding partners. Figure 3B illustrates the electric field’s impact on protomer separation from DNA. At a high simulation E-field (0.4899 V/nm), ASA values approach 4200 Å², close to unbound DNA (4409 Å²). Comparing time-averaged practical E-field (300 V/cm) and no field ASA profiles reveals a significant (*P* ≤ 0.05) 32% increase in DNA surface area with practical E-field. These findings indicate reduced DNA protection at fields as low as 300 V/cm, with higher fields leading to complete DNA-SASP complex dissociation.

### Increased DNA hydration

Figure 3A, B demonstrate electrophoretic unzipping of the DNA-SASP complex, exposing DNA to surrounding chemical agents. Since spore protoplasts contain 28–57% water (wet weight basis)^23^, interactions between DNA and water molecules should be considered in detail. Water molecules, with a small molecular radius ( ≈ 1.4 Å), reach the DNA as the electric field disrupts the SASP protein cover. The radial distribution function (RDF) of water oxygen atoms around DNA was calculated at different FS to assess electric field effects on DNA hydration (Fig. 3C). Higher FS increase water presence in the hydration layer ( ~ 10 Å) around DNA. Supplementary Table 1 shows a statistically significant (*P* ≤ 0.05) increase in water molecules in DNA’s first solvation shell, indicating enhanced DNA hydration due to electric field intensity. This increased hydration can potentially enhance spore killing by facilitating heat transfer to DNA and promoting interactions with hydronium ions (H_3_O^+^), leading to glycosidic bond cleavage.

### Conformational changes under electric fields

Furthermore, the characteristic near-pentagonal shape of the DNA-SASP complex is considered crucial in protecting the DNA against chemical and enzymic attacks^19^. As the DNA-SASP molecule interacts with electric fields, it loses its architecture due to the fluctuations of charged residues, paving the way to access of chemical agents and enzymes to the DNA. Supplementary Movies 1 and 2 illustrate how electric fields affect the DNA-SASP molecule at an atomic level. Supplementary Movie 2 shows increased conformational fluctuations due to the electric field compared to temperature alone in Supplementary Movie 1. RMSD values under varying FS (Supplementary Fig. 2) reveal that higher electric FS lead to greater RMSD values, indicating more significant conformational changes in the DNA-SASP structure. At a high electric field (0.4899 V/nm), the RMSD plot corresponds to vigorous fluctuations seen in Supplementary Movie 2, suggesting substantial disruption of the molecular structure. Comparing RMSD plots between no field and practical electric field (300 V/cm), initial exposure to the electric field slightly reduces RMSD values (≤ 100 ns), but longer exposure (> 300 ns) results in increasing RMSD values, implying potential conformational changes with extended exposure to practical electric fields.

### Discussion and spore inactivation mechanism

An applied treatment can eliminate bacterial spores through various mechanisms, including but not limited to (i) damaging the spore’s DNA, (ii) affecting one or more essential core proteins, (iii) disrupting the germination mechanism of spores, and (iv) compromising the IM of the spore by damaging certain proteins or causing ruptures in the membrane itself^24,25^. Wet heat primarily destroys spores of different *Bacillus* species by damaging one or more vital core proteins^20,25–27^. However, OH introduces additional electrical effects alongside thermal effects, resulting in significantly higher spore killing at the same temperature compared to conductive heating^10^^,^^28–30^. Previous studies on *B. subtilis* spores and their mutants aimed to determine if OH damages spore DNA, RecA protein, or the 2DuF protein in the IM. Results showed no DNA damage at lower EFs, but higher EF assessments were unfeasible due to insufficient spore survivors. The studies indicated that electricity interacts with RecA and IM proteins, though the exact nature of this interaction remains unclear. This study aimed to address unanswered questions from the previous research.

Studies on *B. subtilis* spore mutants revealed that among the tested strains deficient of homologs membrane proteins (YetF, YdfS, and YkjA), YetF had the greatest impact on spore resistance to OH and CH, as shown by similar inactivation profiles in mutants lacking only YetF or all three proteins (Fig. 1B and D). Previous research also highlighted YetF’s major role in spore resistance compared to YdfS^31^, likely due to higher YetF levels relative to YdfS^24^. These proteins belong to the DUF421 family found in the spore IM, which stabilize spore and enhance their resistance to lethal agents^31–34^. Removing SASP and YetF decreased spore resistance even at lower temperatures (Fig. 1C), unlike the SASP-only mutant, which showed increased killing only at higher temperatures^11^. Thus, YetF deletion increases IM permeability, leaving DNA unprotected in the absence of SASP, contributing to increased killing even at lower temperatures with this strain.

The coat slightly reduced spore resistance to both CH and OH, though less than observed with YetF or SASP-deficient spores (Fig. 1E). Coat loss is known to increase core water content and IM permeability, decreasing spore resistance^35^. OH, consistently showed higher killing of decoated spores than CH, indicating no interaction between the electrical component and the coat in rapid spore killing. Removing DPA from the core made spores highly susceptible to treatment, with significant killing observed even at 90 °C for both OH and CH, unlike other mutants. The high DPA level in the core reduces core water content, crucial for spore wet heat resistance^15,36^. DPA removal increased core water content and IM permeability, making spores easier to kill. This eliminated the typical OH-CH inactivation difference at 90 and 100 °C, suggesting electricity interacts with the Ca-DPA complex. At 110 °C, OH killing rates differed significantly from CH, indicating other ohmic effects coming in play at this temperature. Mutants with elevated SpoVA levels showed no change in CH resistance and slightly reduced OH resistance from 90 to 110 °C. However, at 115 °C, OH significantly increased spore killing compared to CH for this mutant (Fig. 1G). This finding is important as it elucidates why a sudden increase in killing is observed at certain temperatures with OH and suggests that with the right combination of temperature and applied EF, SpoVA channels could be opened, facilitating the exit of Ca-DPA from the core, rendering spores much less wet heat resistant. This results in lower core water content and a more flexible IM, leading to higher killing.

Flow cytometry analysis revealed that OH increases the permeability of the IM to PI compared to CH, with the percentage of the spore population stained with PI increasing as the applied EF intensity rises. These results align with our experimental findings, suggesting that OH renders the spore IM more flexible. A similar finding was seen in a separate study noting a higher population of spores stained with PI with OH compared to CH, attributing this to OH potentially disrupting the IM of spores^29^. Thus, higher EF could induce physical damage to the IM, as evidenced by the uptake of PI by 67% of the spore population (Fig. 3), resulting in significantly higher spore killing under these conditions^10,11,14^. Furthermore, molecular dynamics (MD) simulations examining the SASP-DNA dissociation, DNA hydration, SASP’s protein structure, and ASA of DNA, support the conclusion of DNA-SASP dissociation with OH and no such effect with heat alone, with this effect increasing with the rise in EF intensity. This leads to reduced SASP’s protection to the DNA and in particular, enhanced hydration of DNA, facilitating better heat transfer and thus higher killing. Therefore, the higher killing of spores during OH involves a combination of various mechanisms, with some mechanisms coming into play under specific treatment conditions.

**Fig. 4:**
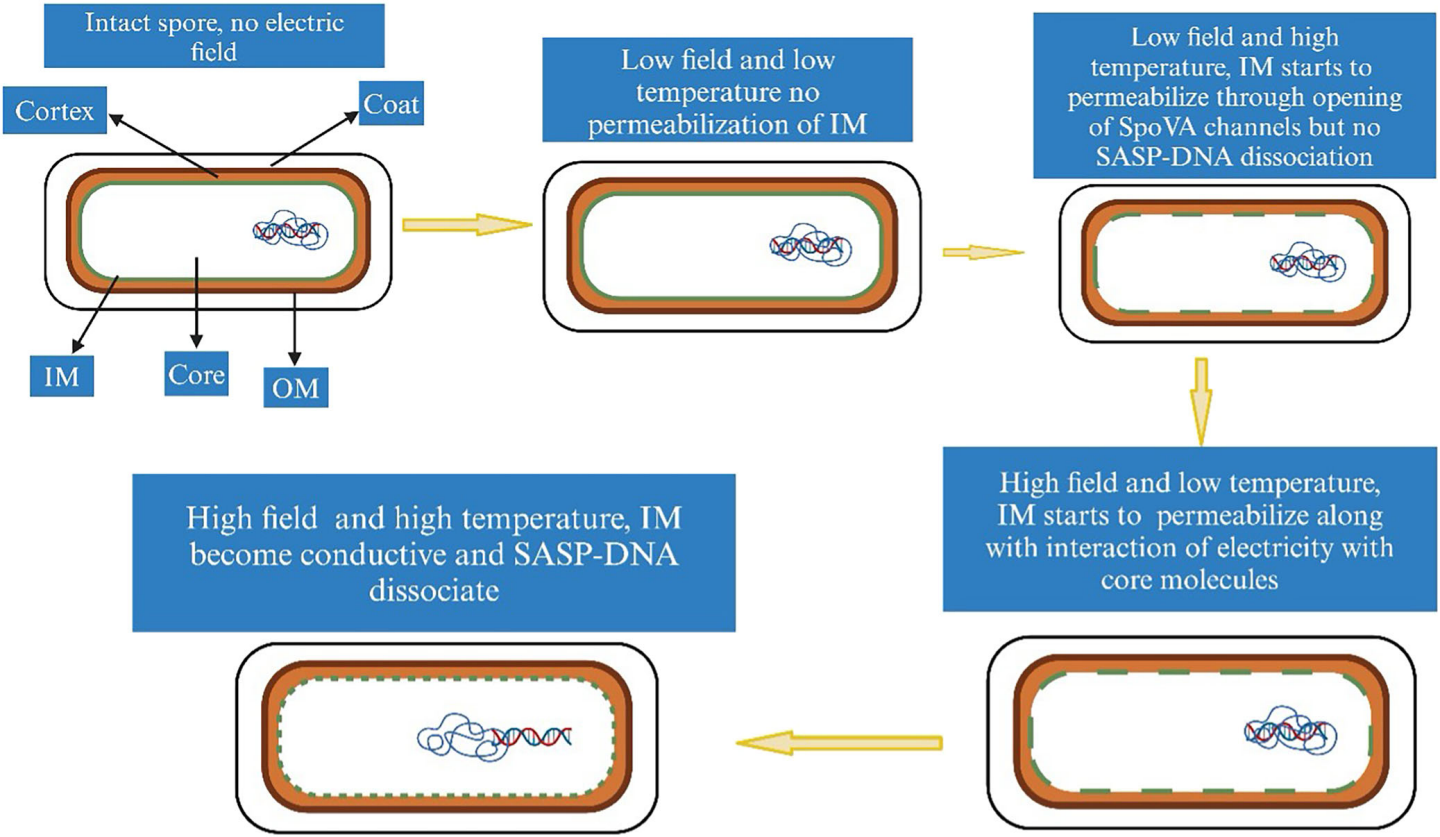
Hypothesized mechanism of spore inactivation during OH At low applied voltage and temperature, the inner membrane (IM) remains impermeable, preventing spore damage. However, applying either high voltage or temperature begins to permeabilize the IM, resulting in increased spore killing. When both high voltage and temperature are combined, the IM becomes conductive by interaction of electricity with key proteins, leading to the loss of the spore’s barrier properties. This allows electricity to dissociate the DNA-SASP complex and interact with other essential core components, ultimately causing complete spore destruction under these conditions.

Based on this and prior work, a summary of the pathways with which OH accelerates spore killing is presented in Fig. 4. OH, introduces non-thermal electrical effects that act in concert with heating to enhance spore killing. At low EF and temperature, the membrane resists electrical passage to the core, explaining why greater killing is not observed under these conditions compared to higher temperatures^10,11,15^. However, as either the applied EF and/or temperature rise, the IM becomes more fluid and conductive, resulting in increased spore killing, as seen both in this work and in another study^29^. Electric fields affect spores by (i) permeabilizing the spore IM and interacting with key IM proteins, and (ii) disrupting the SASP-DNA complex or other core molecules. The first effect is necessary for the second, as ion motion within the spore core requires an entry pathway. This process is similar to electroporation, where electric fields across a bilayer lipid membrane cause membrane compression and failure.

These effects are dependent on treatment conditions, with some effects being observable only at specific temperatures or EF intensities. At higher temperatures, it is found that OH activates the SpoVA channels, resulting in the loss of DPA from the core and increased permeability of the IM (Fig. 1G). Conversely, at higher applied voltages, the forces acting on the spore increase, causing membrane damage even at lower temperatures, which results in significant spore killing^29^. Finally, at higher field intensities and temperatures, maximum killing was observed^10,11^.

MD simulations and results with mutants lacking SASP corroborate these findings, indicating that these intense treatments lead to greater effects on the DNA-SASP structure, along with other effects observed at less lethal conditions. Consequently, these effects result in an increase in the water content of the spore, loss of DNA protection, and ultimately rapid killing of spores during OH. Physics and enzyme studies show electric fields cause three main disruptive effects: linear movement of polar molecules like proteins, oscillatory protein motion due to periodic EF polarity reversal^37,38^, rotational protein response due to dipole moments, and sub-molecular disruptions from attractive and repulsive forces^21^. Therefore, this study enhances our understanding of the interaction between electric fields and spores, laying the groundwork for the design, development, and optimization of an OH process capable of reducing spore levels to a safe threshold with less severe treatment.

## Methods

### OH and CH setup

**Fig. 5:**
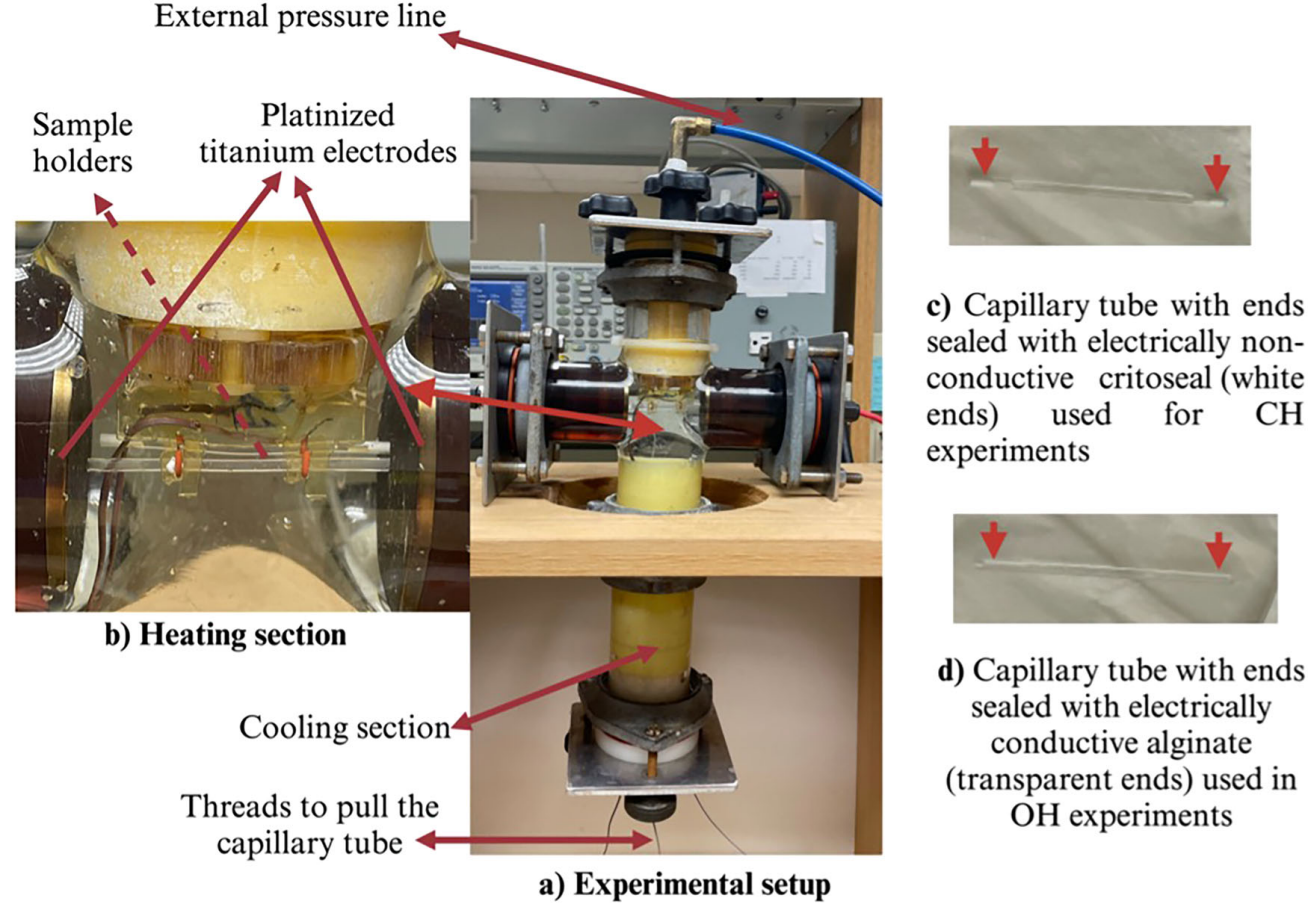
The experimental setup included all the necessary components, with electricity applied using platinized titanium electrodes. **a** Shows the complete experimental setup; the heating section featured a treatment cell in the form of a capillary tube (**b**). Heating section showing electrodes and sample holders. Both OH and CH experiments were conducted in the same chamber, but in separate experiments by simply changing the end plugs of the capillary tube. For CH, the plugs were sealed with an electrically nonconductive sealant (white in Figure **c**), and for OH, they were sealed with electrically conductive alginate (transparent in Figure **d**). The apparatus also had the capability to apply external pressure for experiments conducted at temperatures above 100 °C. Additionally, it included a cooling section filled with an ice-cold solution to rapidly reduce the temperature inside the capillary to below 20 °C.

Both OH and CH experiments were conducted using the same experimental setup (Fig. 5), enabling us to closely match the temperature history between OH and CH. This was crucial for a precise comparison between the two methods, as differing temperature histories yield different inactivation profiles, making the comparison questionable. Supplementary Fig. 3 provides an example of the temperature history, showing that the temperature profiles for all replicate experiments were closely aligned. In all cases (raw data, Singh et al. ^39^), we ensured that CH temperatures were at least equal to, if not greater than those for OH treatments. The setup used was originally developed by Somavat et al. ^14^, and was modified to its current form by Singh et al. ^10^. The setup consisted of samples within capillary cells with either electrically conductive ends (OH) or electrically insulating ends (CH), contained within a larger ohmic heater containing isoconductive fluid. All experiments were conducted at 60 Hz with a bipolar rectangular wave and a 90% duty cycle. The electrodes were powered by a custom-built 20 kW power generator (Model 30,651,074, Semikron GmbH, Nuremberg, Germany). A function generator (Model AFG3252, Tektronix, Inc., Beaverton, OR, USA) was used to adjust the desired waveform, duty cycle, and frequency. The function generator triggered an insulated gate bipolar transistor within the power generator, which acted as a high-frequency switching device to discharge the capacitor accordingly. OH, experiments were conducted at three different temperatures (90, 100, and 110 °C) with a fixed applied field strength of 30 V/cm corresponding to a 165 V RMS. CH experiments were also conducted (in separate experiments) at the same temperature histories as OH 30 V/cm by using a higher field strength (in the range of 165–185 V RMS) to heat the surrounding fluid. All experiments were conducted without a hold time; the samples’ temperatures were raised linearly by applying a constant field strength, and once the desired temperature was reached, the tubes were moved to the cooling section, reducing their temperature to below 20 °C nearly instantaneously. Current (Model 150, Pearson Electronics, Inc., Palo Alto, CA, USA), temperature (T-type thermocouple wire, Omega Engineering, Norwalk, CT), and voltage (high voltage differential probe Model DP10013, Shenzhen Micsig Instruments Co., Ltd., Guangdong, China) data were recorded every second using a datalogger (Agilent Technologies, Santa Clara, CA, USA).

### Bacterial strains used, their preparations

Isogenic derivatives of the *B. subtilis* strain PS832, a prototrophic laboratory version of strain 168, were used. A complete list of the mutant strains employed is presented in Table 1. Spores of all strains were cultivated on 2 × SG medium agar plates at 37 °C without antibiotics, incubated for 2 to 3 days, and then maintained at 23 °C for an additional 2 to 4 days to ensure further cell lysis. Spores were collected and purified based on previously described “Methods”^40,41^. The spore preparations used were at least 98% free from sporulating cells, germinated spores, and cell debris, as confirmed by phase-contrast microscopy. The purified spores from all *B. subtilis* strains yielded 2–4 × 10^9^ spores per plate. These spores were stored in water at 4 °C with an optical density at 600 nm (OD600) of 10–20 and were kept protected from light.

**Table 1:** List of *B. subtilis* mutant spore used

Strain	Details	Reference
PS533	Wild type	Setlow (1996)^25^
PS3411	Overexpress SpoVA operon 4x	Vepachedu & Setlow (2005)^19^
PS4551	SASPless strain lacking YetF	Lab strain Not yet published
PS4550	PS533 lacking YetF	Lab strain Not yet published
PS4531	Lacking YetF, YdfS, and YkjA	Lab strain Not yet published
PS4150	Coatless spores lacking *gerE/cotE*	Ghosh et al. (2008)^36^
PS3406_IN	Lacking SpoVFA and SleB and no DPA in spore core	Mokashi et al. (2020)^16^

### Ohmic and conventional treatment and data analysis

Spores were made in Dr. Peter Setlow’s lab at UCONN Health and shipped to the Sastry lab on dry ice. The initial populations of those spores were 1 × 109 CFU/ml. For both CH and OH treatment these spores were diluted onefold in Na_2_SO_4_ salt solution of defined electrical conductivity (3–3.2 mS/cm). 40 μl of this spore suspension was filled in glass capillary tubes for both OH and CH experiments. The treated suspension was taken out from the capillary tube in a sterile environment, and further diluted in sterile deionized water for enumeration. The spores were enumerated on tryptic soy agar plates, all media used were from (Difco, Becton Dickinson, Sparks, MD, USA), and colony counts were observed for 24–48 h, until it became stable. Ca-DPA (PS3406) deficient spores were incubated for 90 min in 1:1 ratio of 120 mM DPA and CaCl_2_ before plating them on TSA plates. Also, for spores lacking most of its coat, TSA plates were supplemented with 5 μg/ml lysozyme (Sigma-Aldrich, St. Louis, MO, USA). This was done to ensure spore germination after treatment, as decoating removes the cortex-lytic enzyme CwlJ^15^, which is essential for germination. Additionally, this step ensures an accurate estimation of spore inactivation, as spores may not germinate in the absence of lysozyme on the plates, potentially appearing dead and leading to inaccurate calculations^4^. Statistical analysis of the survival data was performed using GraphPad Prism (GraphPad 202 Software, Boston, MA 02110). A two-way ANOVA was conducted, followed by a Tukey test. Statistical significance was determined by a *P* value below 0.05.

### Flow cytometry analysis

We used flow cytometry to assess the impact of our treatments (OH and CH) on the IM of spores. The fluorescent dyes SYTO 16 (Invitrogen, Carlsbad, CA, USA) and PI (Invitrogen, Carlsbad, CA, USA) were employed for this purpose. SYTO 16 can penetrate the membranes of both living and deceased cells, while PI can only traverse the IM if the applied treatment induces structural modifications^42^. The final concentrations of the dyes in the suspension were 15 μM for PI and 0.5 μM for SYTO 16. SYTO 16 was introduced first into the spore suspension and allowed to incubate for 12 min in darkness, followed by PI incubation for 4 min. Consequently, all samples were incubated for 16 min in darkness prior to analysis^43,44^. Flow cytometry analyses were performed using a Cytek Northern Lights cytometer (Cytek Biosciences, Fremont, CA, United States). The green fluorescence of SYTO16, was induced by a 488 nm continuous wave laser at a power of 55 mW and collected through a 525/17 band-pass filter. The red fluorescence of PI was induced by a 488 nm continuous wave laser at a power of 55 mW and collected through a 660/17 band-pass filter. Cheap Sheath (Phoenix Flow Systems, San Diego, CA, United States) was used as sheath fluid. Sample acquisition was carried out at “low” sample flow rate (approximately 20 μl/min). Data were acquired using Cytek SpectroFlo software (Cytek Biosciences, Fremont, CA, United States). A total of 8000-10000 events were recorded for each sample. The unmixed data containing all the fluorescence information from each fluorescent tag in the experiment was analyzed using FlowJo software (FlowJo LLC, Ashland, OR, United States). The same gating strategy was used for all the samples as described elsewhere^44^. Stained and untreated control samples were used to set the gating position.

### Molecular dynamics (MD) Simulation model preparation

We conducted MD studies aimed to investigate the effect of electric fields on the DNA-SASP molecular structure within the spore core. Since MD simulations are extremely computationally intensive, it is common practice to use sufficiently high FS, on the order of 1 V/nm, to capture relevant MD within short timescales, typically in the nanosecond range^22,45,46^. It is known that these FS cannot be applied in practice, but they provide valuable insights that could not be otherwise obtained. In the present study, the field strength of 4.2426 × 10^−^⁵ V/nm, corresponding to an amplitude of 300 V/cm (sine wave), was designated as the ‘practical E-field’ because it could be experimentally applied in ohmic treatments^31^. MD simulations are typically conducted under isothermal conditions to maintain system stability. Here, all simulations were performed at 50 °C, which approximately corresponds to the midpoint temperature of our experiments.

Following steps were followed for simulation model preparation- Step 1: A structure of a DNA-binding α/β-type small acid-soluble protein (DNA-SASP) complex was obtained from the Protein Data Bank (identification code 2Z3X)^19^. The DNA-SASP molecule was parameterized by employing the ff14SB and OL15 force fields available for protein and DNA, respectively. The molecule was solvated with TIP3P water molecules in a box, setting the minimum distance between any solute (DNA-SASP) atom and the edge of the box at 3 Å. Since the diameter of a water molecule is about 2.8 Å, the above solvation method ensures that the entire DNA-SASP molecule was hydrated at least with a monolayer of water. Electrical charges in the hydrated molecule were then neutralized by adding 17 sodium ions (Na^+^) into the water box, which was 70.4 × 70.4 × 69.4 Å^3^ in size. This initial simulation box was used as the core in the model preparation in Step-3.

Step-2: Dipicolinate ion (DPA^2−^) structure information was obtained from the Automated Topology Builder web server (Molecule ID # 10357)^47^. This ion was parameterized with the gaff2 force field recommended for organic molecules like ligands. A calcium ion (Ca^2+^) was created using the tLEaP program within AMBER software, and its interactions were described by 12-6-4 Lennard-Jones-type nonbonded ion parameters, which consist of only electrostatic and van der Waals interactions^48^. Then, a unit containing a 1:1 ratio of Ca^2+^ and DPA^2-^ ions was constructed by enclosing both the DPA^2-^ and the Ca^2+^ ions within a cuboid box (10.4 × 10.8 × 4.0 Å^3^). This unit/structure (denoted as CaDPA) was set as a “solvent” needed in the model preparation (Step-3). Step 3: A new simulation box was constructed by encompassing the initial simulation box (the core, Step-1) and by solvating with CaDPA (Step-2) and TIP3P water molecules. The amounts of these solvating agents were controlled to adjust the density of the final simulation model to be 1.35 g/cm^3^, which is the wet density (at 5 °C) of the calcium-mineralized protoplast of *B. subtilis* spores reported by Beaman & Gerhardt (1986)^49^. The final dimensions of the simulation box were approximately 84 × 84 × 84 Å^3^. The composition (on mass basis) of this simulation model comprised 45.6% of water, 24.8% of CaDPA, and 29.6% of DNA-SASP plus Na^+^ ions.

Prior to MD simulations, the above simulation model underwent preparation using the protocol outlined by Roe & Brooks^50^. First, it was subjected to 300 steps of steepest-descent energy minimization with application of a mild restraint (1.0 kcal mol^−1^ Å^−2^) on DNA-SASP heavy atoms. This minimization was followed by gradual heating to a target temperature of 50 °C over 35 ps in an NVT ensemble, with the same positional restraint on the DNA-SASP heavy atoms. Next, the heated system was subjected to unrestrained equilibration at 50 °C for 15 ps. Subsequently, the system was further equilibrated in an NPT ensemble (50 °C, 1 atm) for 250 ps to attain a stable initial configuration. The equilibrations were performed at a 0.5 fs time step while regulating the temperature and pressure by Langevin thermostat Berendsen barostat, respectively. An 8 Å cutoff was used for treating nonbonding interactions, and long-range interactions were included using the particle mesh Ewald method combined with periodic boundary conditions. Upon the completion of equilibration, production MD simulations were performed for 500 ns at 50 °C and 1 atm while applying a range of FS along the *z*-axis. Three independent simulations (*n* = 3) were performed at each field strength, and trajectories were recorded every 50 ps with 10,000 frames. All the MD simulations and trajectory analysis were carried out with the AMBER 18 software package^51^ available on HPC clusters at the Ohio Supercomputer Center (Columbus, OH, USA). Electric field strength (FS) used were no field, 4.2426 × 10^−5^, 0.1960, 0.4899 V/nm referred in this manuscript as thermal-only (control), practical E-field (300 V/cm), low simulation E-field, and high simulation E-field, respectively.

Analysis of the trajectories was performed using AMBER CPPTRAJ tools^52^. Root mean square deviation (RMSD) values were calculated relative to the first frame of each simulation. The cut-off angle was set to 120°, and the cut-off distance was kept at 3.5 Å (between H-bond donor and acceptor atom) to calculate all hydrogen bonds (H-bonds). Secondary structure content of the SASP protein was assessed with the DSSP algorithm^53^. Intermolecular distance between the DNA and SASP was measured with respect to the center of mass of each molecular type. ASA of the DNA was estimated using the LCPO algorithm^54^ with a probe radius of 1.4 Å. The hydration of DNA was determined by calculating the RDF of the water oxygen atom around the DNA and the number of water molecules in the DNA’s first solvation shell. The PyMOL molecular graphics system (version 2.1.0, Schrödinger, LLC) was used to generate animations of the MD trajectories.

## Supplementary information

Supplementary Information

Movie 1

Movie 2

## Data Availability

Data relating to this study have been deposited as Ag Data Commons Dataset: 10.15482/USDA.ADC/27317316.

## Code availability

The underlying code for this study is not publicly available but may be made available to qualified researchers on reasonable request from the corresponding author.
