# Supplementary material for: Pathways for accelerated bacterial spore killing with ohmic heating
Source: NPJ Sci Food. 2025 Aug 7;9:167. doi: 10.1038/s41538-025-00537-1 (PMC12332034; doi:10.1038/s41538-025-00537-1)
Supplement: Supplementary file 1 — Supplementary Information [file 41538_2025_537_MOESM1_ESM.pdf]

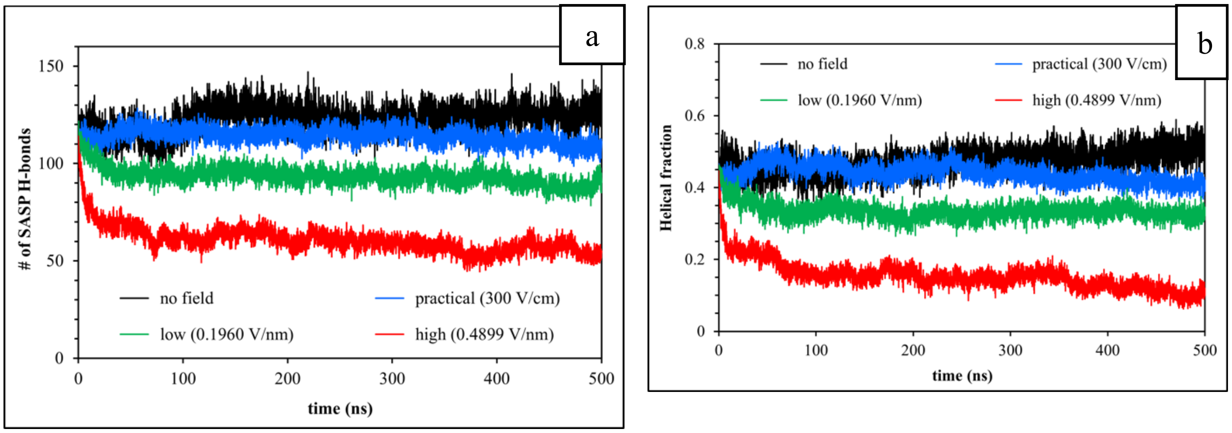

Supplementary Figure 1: Number of intramolecular hydrogen bonds (a), and helical fraction (b) of the SASP protein in the absence and presence of different electric fields. Fig. s1a demonstrates that applying electric fields reduces the number of internal H-bonds in SASP, potentially leading to protein unfolding, with higher field strengths causing greater unfolding. Each protomer of SASP (SASP1, 2, and 3) has two helical segments connected by a turn region, forming a helix-turn-helix motif that binds DNA[17]. Fig. s1b shows the helical fraction of the SASP protein, indicating that higher electric fields correlate with a loss of H-bonds and unwinding of these helical segments.

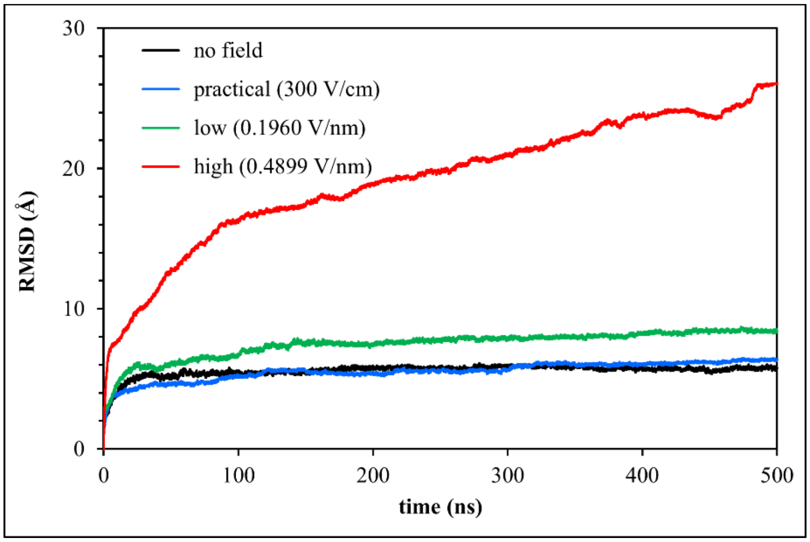

Supplementary Figure 2: RMSD values (for non-hydrogen atoms) of the DNA-SASP structure in the absence and presence of different electric fields. The results also represent stable simulations, except at the high (0.4899 V/nm) electric field.

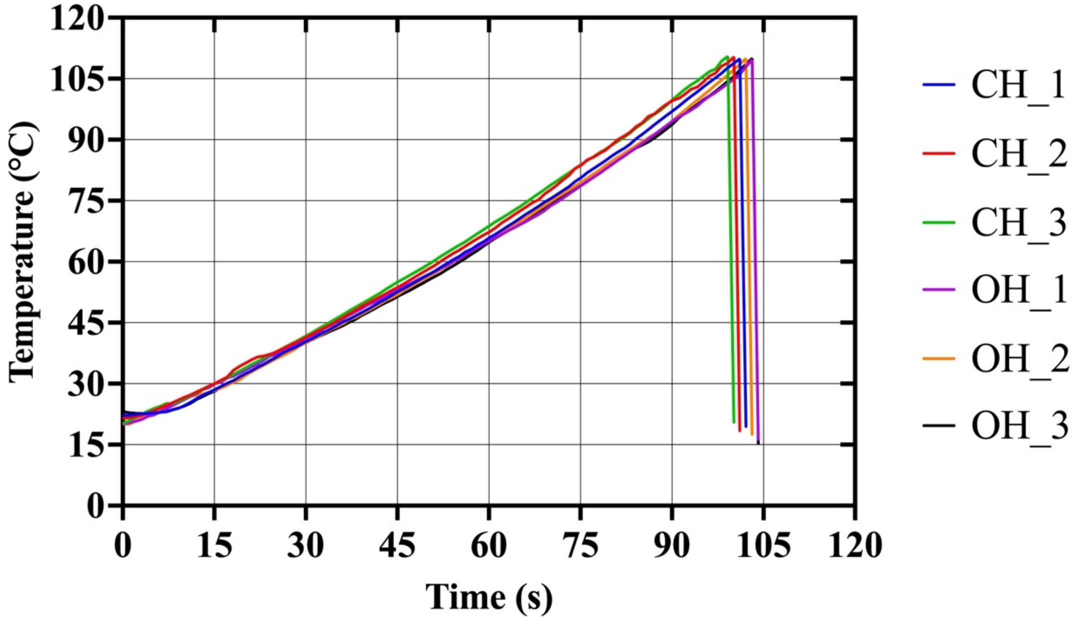

Supplementary Figure 3: shows an example of the temperature evolution over time for replicate experiments using OH and CH at an applied field strength of 30V/cm. To align the CH experiments with OH, a slightly higher voltage was applied in CH. The subscripts 1, 2, and 3 represent the three replicate experiments for both OH and CH, where OH stands for Ohmic heating and CH for Conventional heating.

Supplementary Table 1: Number of water molecules in the DNA's first solvation shell in the absence and presence of different electric fields. Time-averaged values with standard deviations in parentheses are presented.

Electric Field	# of water molecules[a-c]	% increase (w,r,t. no field)
no field	297[a] (18)	0
practical (300 V/cm)	331[ab] (13)	12 (4)
low (0.1960 V/nm)	338[b] (8)	14 (3)
high (0.4899 V/nm)	400[c] (11)	35 (4)

a-c Different superscript letters indicate significant differences ($P \leq 0.05$, one-way ANOVA with Tukey's multiple comparison test).

Supplementary movie 1: The movie depicts the dynamics of the DNA-SASP complex at 50 °C without an electric field (no field). The length of the movie represents the first 15 ns of simulation time at the frame rate of 30 fps. DNA strands: orange; SASP protein shown in the atomic structure model, wherein the spheres represent the atoms (**C**, brown; **H**, white; **N**, blue; **O**, red; **S**, yellow).

Supplementary movie 2: The movie depicts the dynamics of the DNA-SASP complex in the presence of an electric field at 50 °C. All the information is the same as that of S1, except for the applied electric field (= 0.4899 V/nm, Table 1). Observe the deformation of the DNA strands and the fast motion of the SASP atoms that are trying to move away from the DNA strands.
